# Re-Valuation of the Taxonomic Status of Species within the *Inocybe similis* Complex

**DOI:** 10.3390/jof9060679

**Published:** 2023-06-16

**Authors:** Francesco Dovana, Ditte Bandini, Ursula Eberhardt, Ibai Olariaga, Enrico Bizio, Giuliano Ferisin, Fernando Esteve-Raventós

**Affiliations:** 1 Independent Researcher, 15029 Solero, Italy; 2 Independent Researcher, Panoramastraße 47, 69257 Wiesenbach, Germany; ditte.bandini@gmx.de; 3 Staatliches Museum für Naturkunde Stuttgart, Rosenstein 1, 70191 Stuttgart, Germany; ursula.eberhardt@smns-bw.de; 4 Department of Biology and Geology, Physics and Inorganic Chemistry, Rey Juan Carlos University, C/Tulipán s/n, Móstoles, 28933 Madrid, Spain; ibai.olariaga@urjc.es; 5 Società Veneziana di Micologia, c/o Museo di Storia Naturale di Venezia, Fontego dei Turchi, S. Croce 1730, 30135 Venezia, Italy; enrico.bizio@gmail.com; 6 Associazione Micologica Bassa Friulana, Via Vespucci 7, 33052 Cervignano del Friuli, Italy; gferisin@gmail.com; 7 Botany Unit, Department of Life Sciences, University of Alcalá, Alcalá de Henares, 28805 Madrid, Spain; fernando.esteve@uah.es

**Keywords:** Inocybaceae, *Inocybe vulpinella*, phylogeny, taxonomy, sandy soil

## Abstract

The taxonomy of *Inocybe similis* and closely allied species is addressed using morphological and molecular data (nrITS and nrLSU DNA). The holotypes of *I. chondrospora* and *I. vulpinella* and the isotype of *I. immigrans* were studied and sequenced. Our results suggest the synonymy between *I. similis* and *I. vulpinella* as well as that between *I. chondrospora* and *I. immigrans*.

## 1. Introduction

The family Inocybaceae includes about 1050 species and represents one of the species-richest families within the Agaricales [1]. The ectomycorrhizal fungi of the family Inocybaceae are common poisonous mushrooms and are sometimes confused with edible mushrooms. The latter often leads to poisoning [2]. These poisonings are associated with the fact that members of this family are producers of different toxic substances (muscarinic, psilocybin, and psilocin) [3,4].

The genus *Inocybe* includes species that show moderate variability of the macro-morphological features but extreme variability of the microscopic features, hence the large number of species described. Several molecular-based studies pointed out that the recognition and identification of the different species is sometimes difficult due to the more or less partial overlap of the features of similar species or in the presence of cryptic species [5,6,7,8].

Here, we focus on *Inocybe similis* Bres. and its lookalikes as an example of the difficulties in recognising species based on morphology alone. Critical issues regarding correct identification of the species within the *I. similis* complex were already highlighted by Einhellinger in 1994 [9]. *Inocybe similis* is a smooth-spored species described from the area near Trento in Italy in 1905 [10]. Since the study of Dovana et al. [11], in which molecular data from the type collection were used to interpret recently collected specimens, this species is now well understood, and its morphological variability was revealed.

According to Kuyper [12], *I. similis* can only be confused with *I. vulpinella* Bruyl. [13], from which it differs by the presence of a cortina, caulocystidia only present in the upper part of the stipe, basidiospores without an obtuse apex, and thinner-walled pleurocystidia.

Kuyper [12] also proposed the synonymy of *I. chondrospora* Einhell. & Stangl [14], *I. vulpinella*, and *I. immigrans* Malloch [15], species described from Germany, Belgium, and Canada, respectively.

In our previous study [11] of the variability on *I. similis,* it was pointed out that the presence of caulocystidia on different positions of the stipe surface is a variable characteristic probably affected by environmental conditions and the development of the velipellis, and the presence of a cortina as reported by Kuyper [12] was not observed in the samples analyzed in Dovana et al. [11]. This observation raised some doubts about the use of these characteristics to separate *I. similis* from *I. vulpinella* and, more importantly, the status of both taxa.

In order to better clarify the relationships between these mentioned taxa and find morphological characteristics useful to distinguish them, the holotypes of *I. chondrospora*, *I. similis*, *I. vulpinella*, and the isotype of *I. immigrans* as well as recent collections were analyzed to better understand the morphological and genetic variability among them. The main aim of this study is to evaluate the synonymies previously proposed by Kuyper [12] for *I. similis* and *I. vulpinella* using a molecular approach based on the study of the nrITS and nrLSU DNA regions.

## 2. Materials and Methods

### 2.1. Morphology

The macroscopic descriptions were based on fresh material collected in Belgium, Germany, Italy, Slovenia, and Spain. Micromorphological features were observed on fresh and dried material; sections were rehydrated in ammonia 10%, water, or KOH (5% aqueous solution) and then mounted in aqueous Congo red, ammoniacal Congo red, aqueous ammonia, or KOH separately. The terminology follows Kuyper [12]. Cystidia were measured without crystals and basidia without sterigmata. A minimum of 30 basidiospores was measured for each collection. Basidiospore dimensions are expressed as (a) b–c–d (e), where (a) = minimum value, b = average—standard deviation, c = average, d = average + standard deviation, and (e) = maximum value. Q indicates the quotient of length and width of the basidiospores in side view.

### 2.2. Molecular Phylogeny

Genomic DNA was isolated from dry fragments of four freshly collected specimens using the CTAB procedure by Doyle and Doyle [16] or DNA extraction method using NaOH as employed by Dovana et al. [17]. The type specimens of *I. chondrospora* and *I. vulpinella* were extracted following the PTB DNA extraction protocol described by Stielow et al. [18] and that of *I. immigrans* with the Qiagen Puregene kit (Hilden, Germany) following the protocol given in Cripps et al. [7]. The nrITS region was amplified with primers ITS1F-ITS1-58SF/ ITS2-ITS4 [19,20,21], the nrLSU region with primers LR0R/LR5-LR7 [22] and both nrITS and nrLSU regions with ITS1F/TW13 [20]. The sequences obtained in this study were checked and assembled using Geneious vs. R 11.1.5 [23] or Sequencher vs. 4.9 (Genecodes, Ann Arbor, MI, USA) and compared to those available in GenBank (https://www.ncbi.nlm.nih.gov/genbank/) (accessed on 28 July 2022) and UNITE (accessed on 28 July 2022) databases (https://unite.ut.ee/) by using the BLASTN algorithm [24]. Our nrITS-nrLSU dataset includes *Inocybe* sequences selected based on (I) previously molecular studies focused on species belonging to section *Marginatae* subsections *Praetervisae* comprising *I. diabolica* and *I. similis* [25,26,27,28,29,30,31] and on species related to *I. vulpinella* sensu auct. [32] (sequences retrieved from clade: Inocybe_III, Inocybe_IV, and Inocybe_V), (II) the results in BLASTN, and (III) the result of nrITS-nrLSU maximum likelihood tree including 250 best BLAST hits of *I. immigrans* and *I. similis* (tree not shown). The list of sequences used in our dataset is given in Table 1.

Sequences were aligned using MAFFT vs. 7.450 [33] with E-INS-i parameters in Geneious vs. 11.1.5. The nrITS and nrLSU alignment were later automatically trimmed using TrimAl vs. 1.3 software [34] with the option “automated1”, as available in PHYLEMON 2.0 [35].

Maximum likelihood (ML) was inferred with IQ-TREE 2 [36]. The best models were selected using ModelFinder [37], and a total of 1000 ultrafast bootstrap (ufb) replicates were used [38]. The Bayesian inference (BI) was performed with MrBayes vs.3.2 [39]. Partition Finder 2 [40] was used to estimate the best partitioning schemes and evolution models for each subset with the Mrbayes option. Newly generated sequences were submitted to GenBank.

### 2.3. Statistical Analyses

Length, width, and Q variability of the basidiospores of the collections studied are represented by using boxplots drawn in R vs. 3.1.2. The Shapiro–Wilk test was used to test the normality of the sporal morphology data. In the absence of a normal distribution, Kruskal–Wallis tests were performed in order to test at 0.05 significance level whether the length, width, and Q of the basidiospores of the different collections have identical data distributions. In the case of significant differences in the Kruskal–Wallis test, a subsequent Duncan–Waller post hoc test was conducted using the “agricolae” package implemented in R [41].

## 3. Results

### 3.1. Molecular Phylogeny

**Figure 1:**
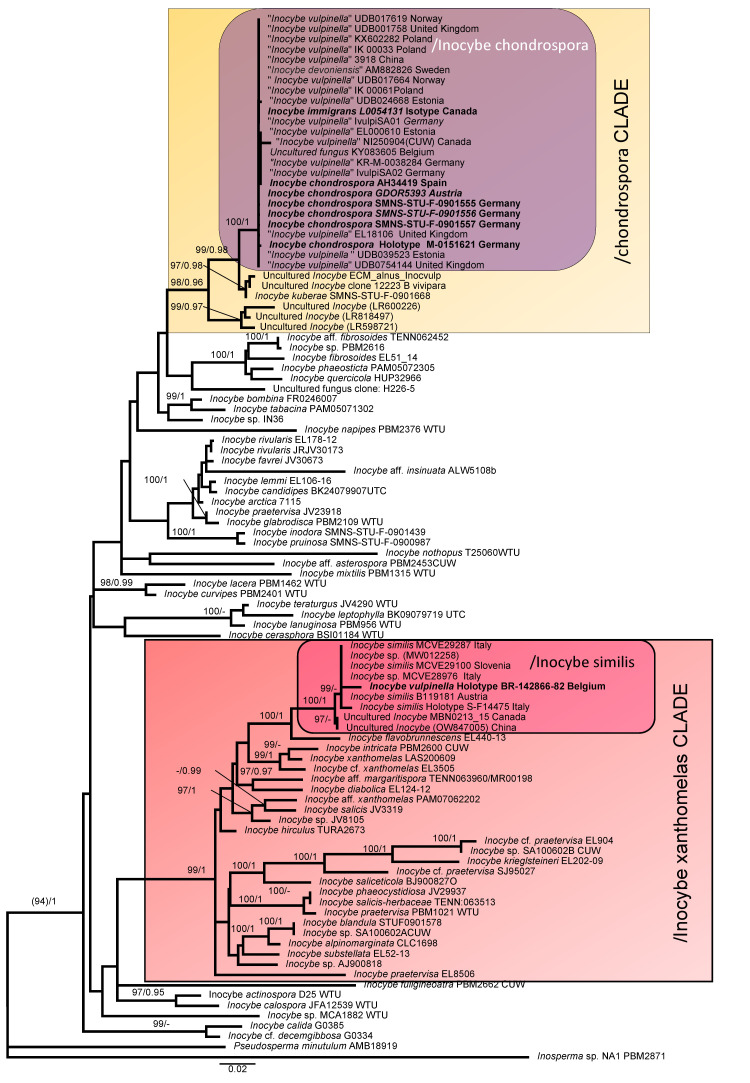
Best tree from the ML analysis of the nrITS + nrLSU dataset. Bootstrap values (ufb) ≥ 95% and posterior probabilities (BPP) ≥ 0.95 are indicated on or below the branches. The GenBank code has been added in brackets when the collection code is not available.

The nrITS and nrLSU DNA combined datasets comprised 1388 characteristics and consisted of 102 sequences. In Figure 1, we present the best tree from the ML analysis of the nrITS + nrLSU dataset, with bootstrap values ≥ 95% and posterior probabilities ≥ 0.95. We found two supported clades that included our sequences: /Inocybe chondropsora (BPP = 1; ufb = 100) in the/chondrospora clade and/Inocybe similis (BPP = 1; ufb = 100) in the /Inocybe xanthomelas clade (Figure 1). The sequences of the holotypes *of I. chondrospora* and the isotype of *I. immigrans* grouped in the same clade (/Inocybe chondrospora) with other 23 sequences, 18 retrieved from GenBank and UNITE databases (17 sequences identified as *I. vulpinella* and a sequence identified as *Inocybe devoniensis*), and 7 sequences newly generated in this study (Figure 1). *Inocybe chondrospora* is sister to *Inocybe kuberae* Bandini & B. Oertel (BPP = 0.98; ufb = 99), and both with other environmental sequences belong to the /chondrospora clade (BPP = 0.96; ufb = 98). Holotypes of *I. similis* and *I. vulpinella* grouped in the same clade (/Inocybe similis: BPP = 1; ufb = 100) with another seven sequences retrieved from GenBank./Inocybe similis is the sister clade of *I. flavobrunnescens* Esteve-Rav., G. Moreno & Bizio, and both are grouped within species belonging to Inocybe section *Marginatae* Kühner and *I. diabolica* Vauras in the/Inocybe xanthomelas clade.

### 3.2. Statistical Analysis

**Figure 2:**
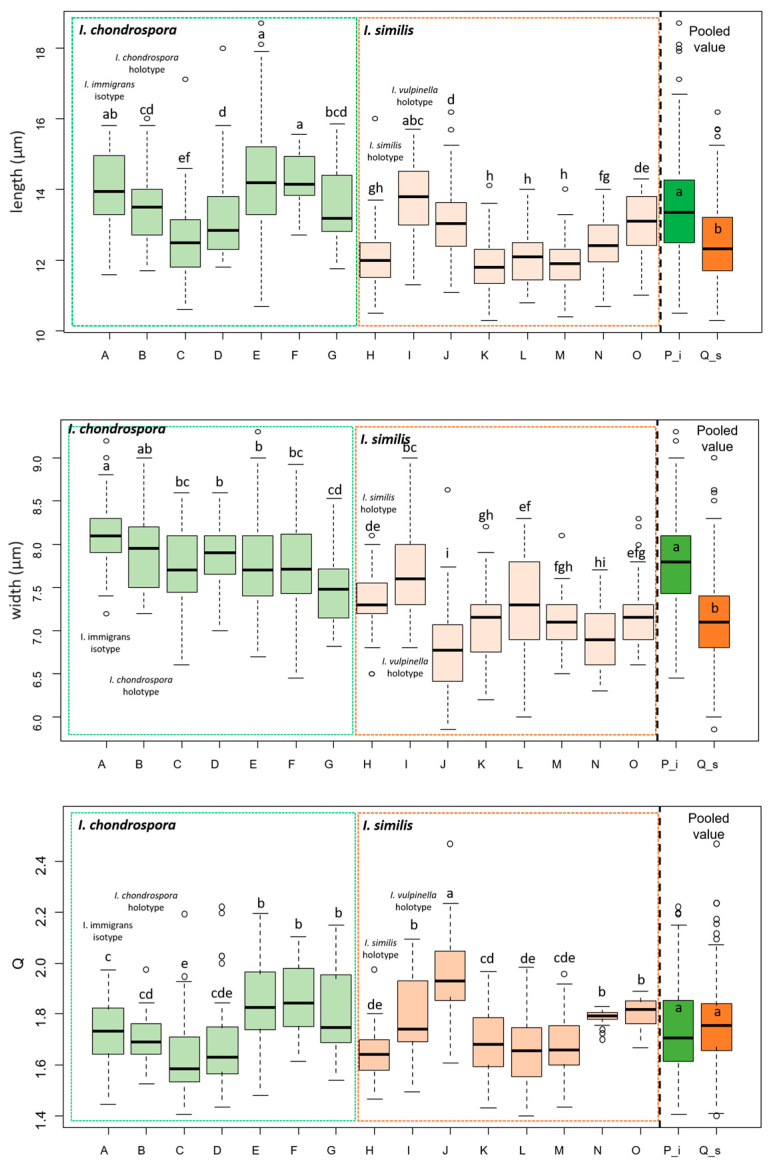
Boxplots comparing length (FIG2A), width (FIG2B), and Q value (FIG2C) of *I. chondrospora* basidiospores (A, *I. immigrans* isotype; B, *I. chondrospora* holotype; C, DB4-5-13-2; D, DB26-9-17-7b; E, DB2-9-12-4; F, AH34419; G, GDOR5393; P_i, pooled value of *I. chondrospora*) and *I. similis* (H, *I. similis* holotype; I, *I. vulpinella* holotype; J, MCVE29100; K, BAN11-9-18-1; L, BAN1718; M, BAN2609; N, MCVE28976; O, MCVE29287; Q_s, pooled value of *I. similis*) basidiospores. The small letters above the boxes indicate significant differences between collections according to Duncan–Waller post hoc test *p* < 0.05.

Analyses of “pooled data” (includes all the measurements of the different collections belonging to the same species) show a larger size of the basidiospores of *I. chondrospora* than those of *I. similis*, while the Q value does not show significant differences (Figure 2). The average basidiospore length of *I. chondrospora* is 13.6 μm, which is 9% greater than the basidiospore average length of *I. similis* (average value: 12.5 μm), while the width average of *I. chondrospora* is 7.8 μm, which is 9% greater than the basidiospore width of *I. similis* (average value: 7.1 μm). The length of the basidiospores of the isotype of *I. immigrans* shows no difference compared to the holotype of *I. vulpinella*, while it is greater than the holotype of *I. chondrospora* and the holotype of *I. similis* (Figure 2); five out of eight collections of *I. similis* (including the holotype of *I. vulpinella*) show a shorter basidiospore length than all the *I. chondrospora* collections, and six out of eight collections examined show smaller widths. Length, width, and Q of the individual collections have significant differences not only between the two species considered in this study but also show significant differences within the same species.

### 3.3. Taxonomy

**Figure 4:**
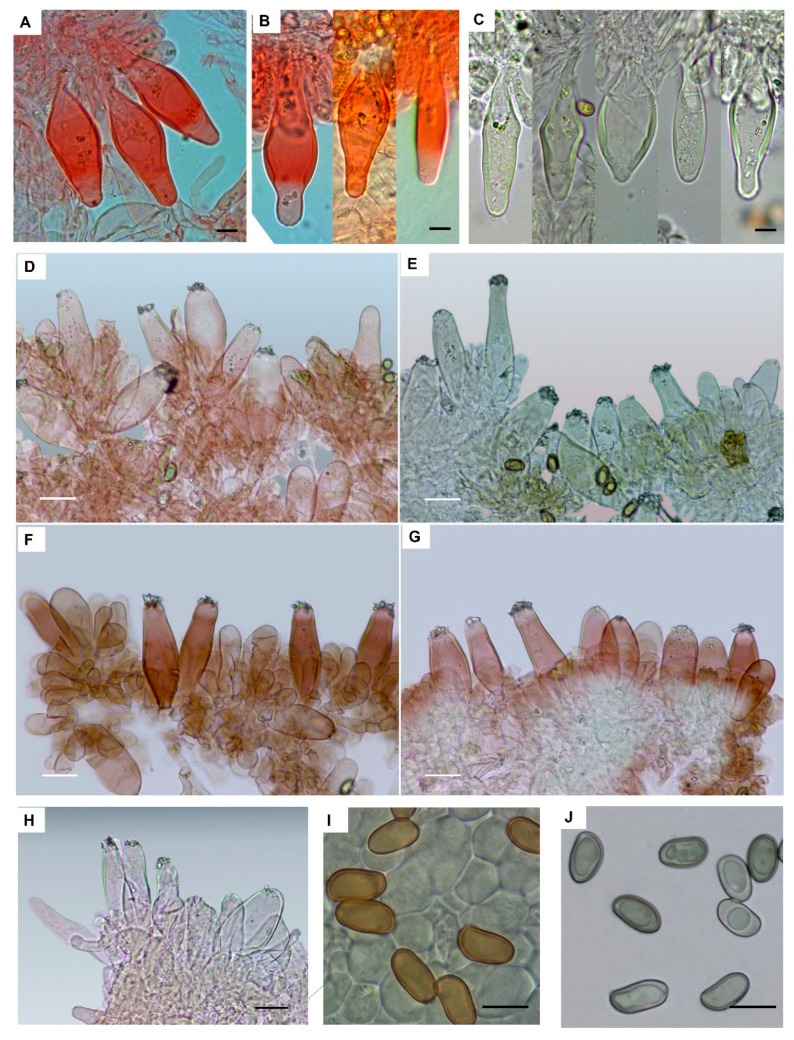
*Inocybe similis*. (**A**,**D**–**G**) Cheilocystidia ((**A**) MCVE29100; (**D**,**E**) MCVE28976; (**F**,**G**) MCVE29287). (**B**,**C**) Pleurocystidia (MCVE29100). (**H**) Caulocystidia (MCVE29287). (**I**,**J**) Basidiospores ((**I**) MCVE29100; (**J**) MCVE29287). Scale bar: (**A**–**C**); (**I**,**J**) 10 µm; (**D**–**G**) 20 µm.

**Figure 5:**
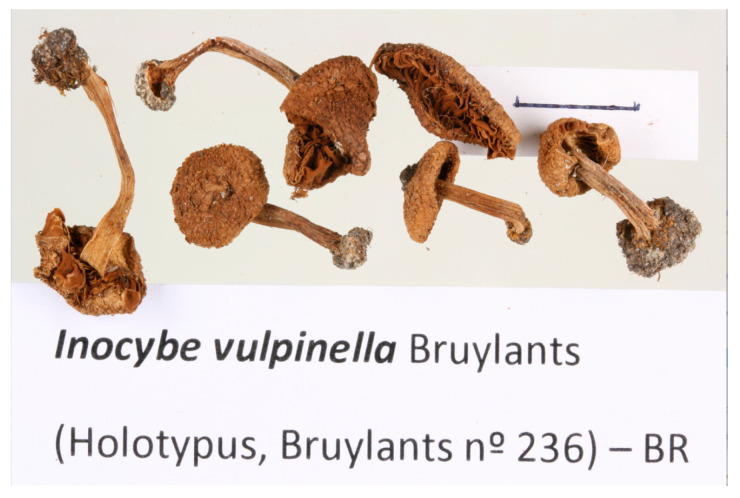
Holotype of *Inocybe vulpinella*. Scale bar: 1 cm.

*Inocybe similis* Bres., Ann. Mycol. 3 (2): 161 (1905) Figure 3, Figure 4 and Figure 5.

= *Inocybe vulpinella* Bruyl., Bull. trimest. soc. mycol. Fr 85: 341 (1970).

The description is based on nine recent collections and the holotypes of *I. similis* and *I. vulpinella*: Pileus 20–35 mm, initially campanulate or hemispherical, then convex, and finally plano-convex, with broad and low central umbo, pileus surface woolly-fibrillose, opaque, dry, from extremely fine to coarsely squamulose and often with quadrangular or fringed squamules or fibre bundles, at margin not rimulose and not striate, ochraceous, yellowish-brown to cinnamon-brown, generally lighter at the disk in younger specimens due to the presence of a greyish-white veil strongly sticking to the cuticle. Cortina not observed. Lamellae moderately crowded, adnate to emarginate, pale cream to ochraceous when young, finally brown to olivaceous-brown, edge fimbriate, whitish. Stipe 50–65 × 4–5 mm, central, firm, solid, generally inserted in the sandy substrate for about one-third of its length, equal to sub-bulbous at base, sometimes ending in a small napiform bulb, usually completely pruinose and sometimes somewhat sparsely in the lower part, probably due to abrasion through sand, sometimes longitudinally striate, whitish when young, often with the middle part ochraceous, then concolourous with pileus. Context fibrous, compact, whitish in the cap, whitish to yellowish in the stipe, smell absent.

Basidiospores (10.5)11.4–12.5–13.6(–16.0) × (6.0–)6.6–7.1–7.7(–9.0) μm, Q = (1.40–)1.61–1.76–1.90(–2.47); smooth, regular to sub-phaseoliform, sometimes with a largely obtuse apex but in several collections subangular. Basidia 30–45 × 10–18 μm, clavate, 4-spored. Pleurocystidia 45–110 × 14–28 μm, highly variable in shape, subcylindrical, narrowly utriform, fusiform or clavate, not markedly lageniform, thick-walled; wall up to 2.0–3.0 μm (rarely up to 5.5 μm in the apex) thick, occasionally very thin in the apical portion, bright yellow or hyaline in KOH solution, generally with crystals at apex. Cheilocystidia 50–70 × 17–25 μm, similar to pleurocystidia. Paracystidia up to 30 × 15 μm, abundant, clavate to pyriform. Caulocystidia present along the whole length of stipe or in upper half only, their distribution over the stipe surface very variable among collections, solitary or mainly in clusters, similar to pleurocystidia but more irregular in size and shape. Few cauloparacystidia present in lower part of stipe. Pileipellis a cutis, hyphae 7–5 m wide; terminal elements clavate; pigment ochraceous, parietal, and intracellular, sometimes slightly encrusting and parietal. Clamp-connections present in all tissues.

Habit, habitat, and distribution: in groups, growing on sandy soil with the presence of species belonging to the families Pinaceae and Salicaceae, present in Asia, Canada, and Europe.

Specimens examined:

Austria. Tyrol, Reutte, Forchach, bank of river Lech, in sand and gravel, with Salix sp., 11 September 2018, leg. D. Bandini (DB11-9-18-1); ibidem, Salix sp., 11 September 2018, leg. D. Bandini (DB11-9-18-4); Tyrol, Reutte, Rieden, Lechaue, ÖK25V 2215-West, alt. 870 m, Salix sp., Pinus sylvestris, 19 September 2018, leg. D. Bandini (DB19-9-18-23).

BELGIUM. Antwerp, “in arena conchyliosa fluminis Escaut”, 18 June 1955, Julia Bruylants n°236 (holotype of *I. vulpinella*) (Figure 5). Measurements conducted on the holotype of *I. vulpinella*. Basidiospores: (11.3–)12.8–13.8–14.8(–15.7) × (6.8–)7.1–7.7–8.3(–9.0) μm, Q = (1.49–)1.63–1.79–1.95 (–2.09). Pleurocystidia: 47–66 × 14–22 μm (average data = 57 × 17 μm).

Germany. Bavaria, Schwaben, Ostallgäu, Füssen, TK25 8430/1, alt. 820 m, shore of river Lech with Salix sp., 22 September 2016, leg. D. Bandini (DB22-9-16-18); ibidem, at some distance from former location, 12 October 2016, leg. D. Bandini (DB12-10-16-19); ibidem, at some distance from the former location, alt. 800 m, Salix sp., 20 September 2018, leg. D. Bandini (DB20-9-18-9).

Italy, Trentino Alto Adige, Trento, place called “desert” by Bresadola, May 1900, G. Bresadola, Holotype F-S14475. Friuli Venezia Giulia, Grado, Municipal park consisting of a flat sand ground, in the presence of *Populus tremula* and *Pinus halepensis*, 13 July 2014, G. Ferisin, MCVE 28976; ibidem, 1 May 2014, MCVE29287.

Slovenia. Goriška, Tolmin, close to the levee of Isonzo river, on alluvial sandy-gravelly soil, near *Populus tremula* and Salix sp., 11 October 2008, leg. E. Bizio and A. Aiardi, MCVE29100.

**Figure 7:**
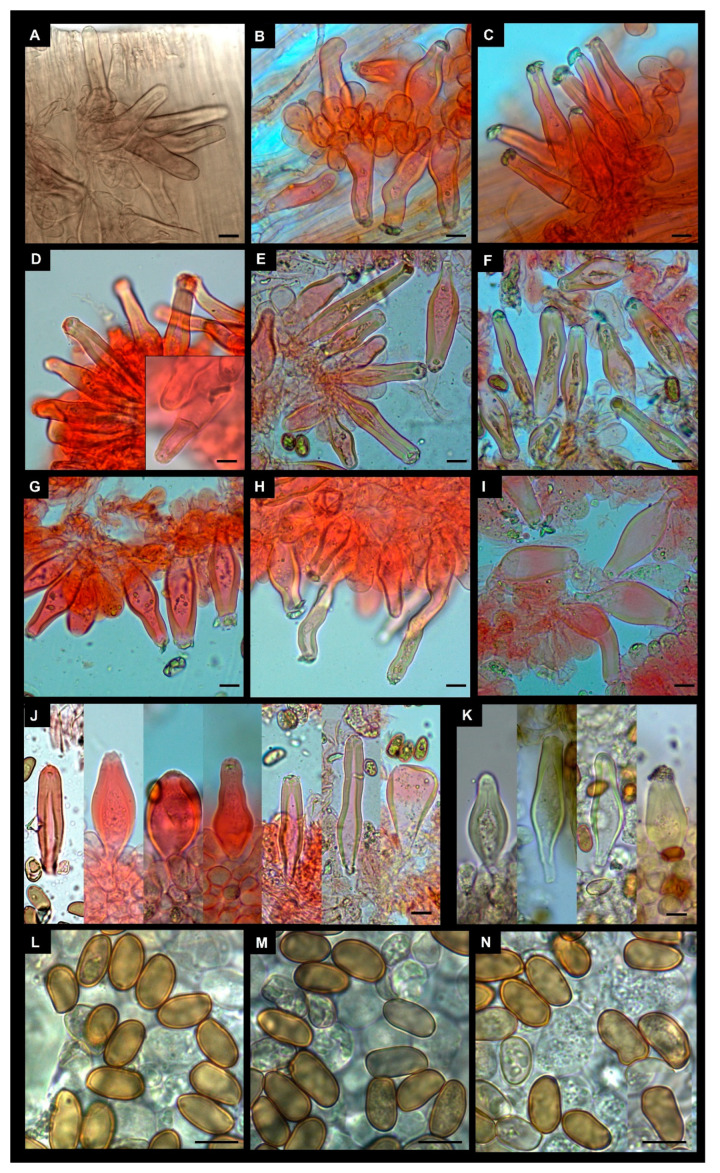
*Inocybe chondrospora*. (**A**–**C**) Caulocystidia (AH 34419). (**D**–**I**) Cheilocystidia ((**D**) GDOR5393; (**E**–**I**) AH 34419). (**J**,**K**) Pleurocystidia (AH 34419 and GDOR5393). (**L**–**N**) Basidiospores ((**L**,**M**) GDOR5393; (**N**) AH 34419). Scale bars: 10 μm.

**Figure 8:**
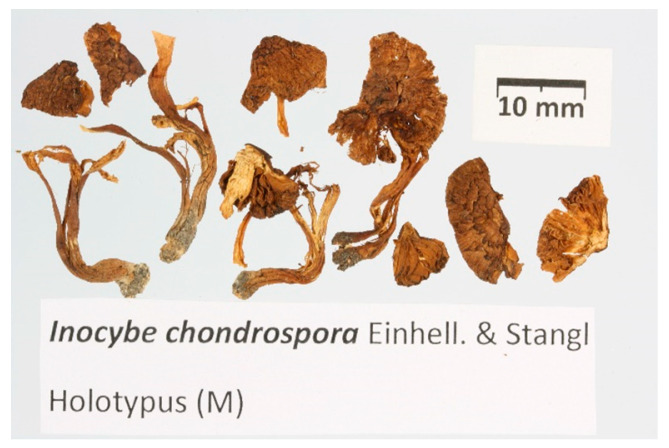
Holotype of *Inocybe chondrospora*. Scale bar: 1 cm.

*Inocybe chondrospora* Einhell. & Stangl, Z. *Mykol.* 45(2): 163 (1979) Figure 6, Figure 7 and Figure 8.

= *Inocybe immigrans* Malloch, *Can. J. Bot*. 60(1): 40 (1982).

The general description is based on five new collections, the holotype of *I. chondrospora*. and the isotype of *I. immigrans*.

Pileus 8–50 mm, initially campanulate or hemispherical, finally convex or plano-convex, sometimes becoming slightly depressed at centre, generally with a broad and prominent central umbo, initially radially fibrillose or rarely nearly glabrous, then conspicuously tomentose-scaly, finally often cracking toward the centre, with scales often becoming dark cinereous or silvery and appearing frosted or brownish orange to greyish or yellowish brown or dark brown when young and fresh, darkening in late stage of maturation through brown to dark brown or reddish brown, generally remarkably lighter at the margin in younger specimens but also sometimes observable in old samples. Lamellae more or less crowded, adnate to emarginate, pale cream, yellowish grey, greyish yellow to ochraceous when young, finally brown to brown olivaceous; edge fimbriate, whitish. Stipe 10–70 × 1.5–9 mm, central, firm, solid, equal or slightly swollen at base to sub-bulbous at base (up to 10 mm), yellowish white when young, then greyish orange to orange-grey, then from light brown to dark brown, at apex minutely flocculose and whitish, often whitish also at the base. Cortina not observed. Context fibrous, compact, whitish to yellowish, rarely darker in the pileus, lacking a distinctive odour.

Basidiospores (10.5–)12.3–13.6–14.9(–19.0) × (6.5–)7.3–7.8–8.3(–9.5) μm, Q = (1.40–)1.57–1.75–1.93(–2.22) variable in shape between the same basidioma and between the different collections, generally smooth, ellipsoid to oblong, rarely subcylindrical, seldom also sub-ovoid, sometimes slightly irregular to rarely slightly gibbous. Basidia 25–50 × 8–18 μm, clavate, four-spored. Pleurocystidia 38–80 × 11–28 μm, highly variable in shape, subcylindrical, narrowly utriform, fusiform, ventricose or clavate, thick-walled up to 6 μm (sometimes filling the whole lumen of the upper, narrow part of the cystidia), bright yellow or light yellow in KOH solution, generally crystalliferous at apex. Cheilocystidia similar to pleurocystidia but more variable in shape even in the same basidioma (see Figure 7E–I), sometimes fusiform with long narrow necks, scattered to abundant, sometimes septate, thick-walled; wall up to 6 μm thick, often of equal thickness over the whole length of the cystidia. Caulocystidia present along the whole length of the stipe, less abundant in the lower half, partly similar to cheilocystidia, partly more irregular in shape (Figure 7A–C), thick-walled with walls up to 5 μm thick, mixed with cauloparacystidia. Pileipellis a cutis, 8–15 µm wide, with clavate terminal elements, pigment ochraceous, parietal, and intracellular, sometimes indistinctly encrusted. Clamp connections present.

Habit, habitat, and distribution: in groups, growing on sandy soil with presence of species belonging to the families Betulaceae, Orchidaceae, Pinaceae, and Salicaceae, present in Asia, Canada, and Europe.

Specimens examined:

Austria. Similaun, Rotmoos, sandy soil in association with Salix herbacea, leg. E. Bizio, GDOR5393; CANADA. Ontario, Hastings Co., Faraday Township, Bow Lake, Madawaska Mines, 16 June 1979, leg. D. Malloch (isotype of *I. immigrans*) (L0054131). Measurements conducted on the isotype of *I. immigrans*. Basidiospores: (11.5–)13.0–14.0–15.1(–1.8) × (7.0–)7.7–8.2–8.6(–9.2) μm, Q = (1.44–)1.59–1.72–1.85 (–1.97). Pleurocystidia: 38–65 × 14–19 μm (Average data = 52 × 16 μm).

Germany. Bavaria, Murnauer Moor, MTB 8333, 10 Km west, 31 May 1966, leg. A. Einhellinger M-0151821 (holotype of *I. chondrospora*). Measurements conducted on the holotype of *I. chondrospora*. Basidiospores: (11.5–)12.5–13.5–14.5(–16.0) × (7.0–)7.5–7.9–8.4(–9.0) μm; Q = (1.52–)1.61–1.70–1.79 (–1.95). Pleurocystidia: 45–68 × 14–22 μm (average data = 55 × 17 μm). Rheinland-Pfalz, Rhein-Pfalz-Kreis, Rheinauen, Altrip, TK25 6516/4, alt. 90 m, sandy soil with Salix sp., *Populus tremula*, 4 May 2013, leg. D. Bandini & B. Oertel (DB4-5-13-2); Schleswig-Holstein, Ostholstein, Malente, Sieversdorfer Kiesgrube, TK25 1729/3, alt. 50 m, sandy soil with *Populus tremula*; Salix sp., *Pinus sylvestris*, Betula sp., 26 September 2017, leg. G. Schmidt-Stohn & B. Oertel (DB26-9-17-7b).

Netherlands, Friesland, Ameland, Hollum, alt. 0 m, white dunes with *Salix repens*, 2 September 2012, leg. D. Bandini (DB2-9-12-4); SPAIN. Cantabria, Liencres, Piélagos, 10 October 2004, littoral sand dunes with presence of *Salix* cf. *repens*, leg. F. Prieto & M.A. González, AH 34419 (in [42], as *I. vulpinella*).

## 4. Discussion

The phylogenetic analyses of the ITS and LSU data from type collections in the *I. similis* group showed the conspecificity between *I. similis* and *I. vulpinella* and confirmed that *I. chondrospora* and *I. immigrans* belong to a single taxon not related to *I. vulpinella*.

Several authors have considered *I. similis* and *I. vulpinella* as independent species that are separable based on macroscopic and microscopic characteristics [12,43]. Both Kuyper [12] and Stangl [43] placed *I. similis* into the “supersection cortinatae” and *I. vulpinella* within the “supersection marginatae” based on the presence/absence of cortina and distribution of caulocystidia. However, Kuyper [12] did not point out “Marginatae” and “Cortinatae” as formal taxonomic-level units. Neither were the presence of a cortina nor the arrangement of the caulocystidia on the stipe mentioned by Bresadola [10] in his original description of *I. similis*; however, in more recent morphological classifications, these are two fundamental characteristics [12,43].

Moreover, differently from Kuyper’s opinion, those *I. similis* collections examined by Dovana et al. [11] did not have a cortina and showed a variable distribution of the caulocystidia on the stipe in the different collections. This last characteristic is often influenced by the development of the velipellis. Kuyper stated that *I. vulpinella* is morphologically closest to *I. similis*, although it must belong to a different “supersection”. He also noted that the former species differs from *I. similis* in that its basidiospores show an applanate apex and thicker-walled pleurocystidia.

The molecular analysis suggested that these differences represent variability within the same species. The basidiospore measurements reported in Kuyper for *I. vulpinella* (12.0–18.0 × 7.0–9.0 μm) are larger than those of *I. similis* (11.5–16.0 × 7.0–8.5 μm), even though this has not been considered by the same author as a characteristic to distinguish the two species from each other [12]. Our statistical analyses of the basidiospore measurements showed that the basidiospores of the holotype of *I. vulpinella* are significantly larger than those of the holotype of *I. similis* and the other *I. similis* collections (Figure 2). In this study, we did not consider *I. vulpinella* var. *fuscolamellata* Bon, which is a taxon described by Marcel Bon and considered a synonym of *I. vulpinella* [12]; however, the possible conspecificity of this taxon with *I. similis* or *I. chondrospora* would not have priority at the species rank.

The molecular analysis supported the synonymy of *I. chondrospora* and *I. immigrans*, but unlike what Kuyper [12] considered, our phylogenetic analyses place both in a lineage independent and distant from *I. similis/vulpinella*.

*Inocybe chondrospora* is found to be the oldest name for this taxon, as it was published and used in 1979, three years before *I. immigrans*. Sixteen sequences that grouped in the /chondrospora clade were erroneously identified as *I. vulpinella*. This fact is not surprising considering that these three species have been accepted as synonymous by most mycologists since Kuyper [12].

In the same clade, there is a sequence from Sweden (GenBank: AM882826 [25]) named “*Inocybe devoniensis*”. *Inocybe devoniensis* P.D. Orton [44] shares some features (for example, large basidiospores of comparable size) with *I. chondrospora*, but it differs mainly in the presence of (sub)rimose, smooth pileus, sub-lageniform cystidia with much thinner wall, typically ellipsoidal basidiospores, and caulocystidia only in the upper part of the stipe [12,44]. The slightly angulose outline of the basidiospores has not been observed in the study of *I. devoniensis* either. These differences were also confirmed in the microscopic analysis conducted on the *I. devoniensis* holotype by one of us (Esteve-Raventós, unpublished data).

Subsequent molecular studies on the holotype of *I. devoniensis* will be required to definitively clarify its position; if it is found to be the same taxon as *I. chondrospora*, the name *I. devoniensis* would have priority, but this possible conspecificity is unlikely.

In the microscopy table attached to the protologue of *I. chondrospora,* a caulocystidium with an intermediate septum is presented, although this characteristic is not reported in the text. Septa within cheilocystidia and pleurocystidia are not unusual in *I. chondrospora* (see Figure 7). Although it does not appear to be a constant characteristic, this type of septum has not been observed in *I. similis* and could be an additional characteristic to separate the two species.

A remarkable feature of *I. chondrospora* is the great variability of the basidiospore dimensions both within the individual basidiomes and among the different collections. In the original description of *I. chondrospora*, Einhellinger and Stangl reported values of 11–19 × 8–10 µm for the holotype [14], and Malloch, when describing *I. immigrans*, indicated pooled data values of (8.9–)10.0–16.0(–20.0) × (6.0–)6.5–8.5(–9.8) µm, and for single collections, the average basidiospore length was reported as 10.9–14.9 µm [15]. Summarising, the differences between *I. similis* and *I. chondrospora* are not always well defined in the single collections, and it is also possible to find basidiomata with intermediate characteristics. In most of the studied collections, the basidiospores of *I. chondrospora* have larger dimensions than those of *I. similis* even if the basidiospore dimensions of some collections (e.g., basidiospores of *I. vulpinella* holotype) are similar to those of *I. immigrans*. In conclusion, *I. chondrospora* and *I. similis* represent two species with similar variability in morphological characteristics and distribution areas (Asia, Canada, and Europe). They are also associated with the same plants, mainly in habitats where Pinaceae and Salicaceae are present, although *I. chondrospora* can also associate with *Liparis loeselii* (Orchidaceae) and probably some Betulaceae (see Table S1). In typical collections of both species, (I) the pileus surface is rougher scaly with larger fibre bundles in *I. similis* compared to *I. chondrospora*; (II) usually in *I. similis*, there is no such colour contrast in the pileus, as is often observed with *I. chondrospora*; (III) generally, there are smaller basidiospores in *I. similis* (11.4–12.5–13.6 × 6.6–7.1–7.7 μm) versus *I. chondrospora* (12.3–13.6–14.9 × 7.3–7.8–8.3 μm); (IV) there are longer hymenial cystidia in *I. similis* (pleurocystidia 45–110 × 14–28 μm) versus *I. chondrospora* (pleurocystidia 38–80 × 11–28 μm) (average values of pleurocystidia in German collections: 67 × 19 µm in *I. similis* vs. 55 × 16 µm in *I. chondrospora*), and (V) there are thicker-walled cystidia in *I. chondrospora*.

In the case of non-typical collections (for example, in the holotype of *I. vulpinella*), which have intermediate characteristics, the best way to tell the species apart is to use DNA data even considering that they can share a similar habitat.

## Figures and Tables

**Figure 3:**
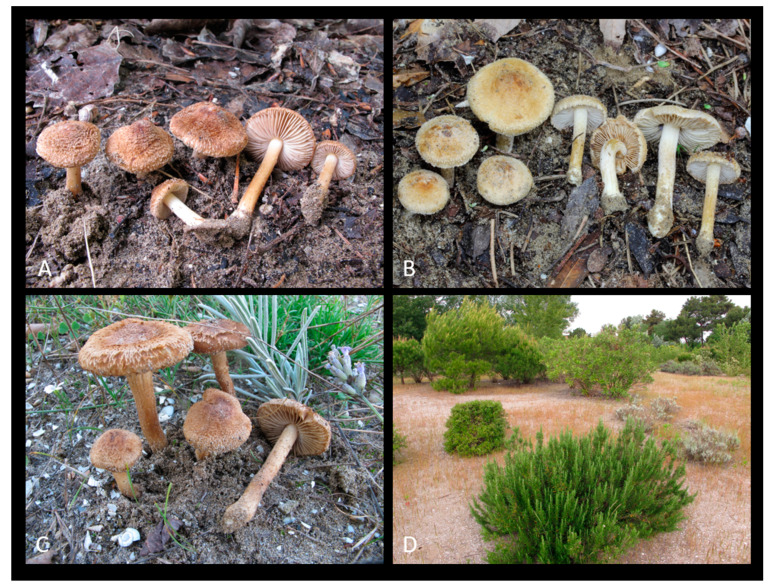
(**A**–**C**) Fresh basidiomata of *Inocybe similis* ((**A**) MCVE29287; (**B**) MCVE 28976; (**C**) MCVE29287). (**D**) Italy, Grado, where MCVE28976 and MCVE29287 specimens were collected.

**Figure 6:**
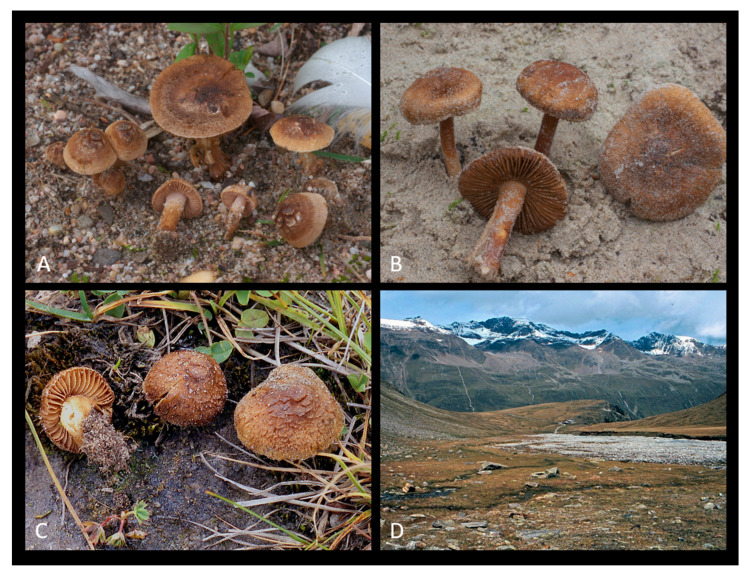
(**A**–**C**) Fresh basidiomata of *Inocybe chondrospora* ((**A**) DB4-5-13-; (**B**) DB-2-9-12-4; (**C**) GDOR5393); (**D**) Austria, where the GDOR5393 specimen was collected.

**Table 1:** *Inocybe* collections used in phylogenetic analyses. Sequences in bold and with * were newly generated for this study, ** sequences belonging to /Inocybe chondrospora, and *** sequences belonging to /Inocybe similis.

Taxon	Collection	Country	GenBank/UNITE	
			ITS	nLSU
*Inocybe actinospora*	D25 WTU	Argentina	X	AY380363
*Inocybe alpinomarginata*	CLC1698	USA: Colorado	MK153647	MK153647
*Inocybe arctica*	7115	Sweden	KY033839	KY033839
*Inocybe* aff. *asterospora*	PBM2453 CUW	Unknown	X	AY702015
*Inocybe blandula*	SMNS-STU-F-0901578	Austria	MZ144124	MZ144124
*Inocybe bombina*	FR 0246007	Germany	NR_173842	X
*Inocybe calida*	G0385	Hungary	MK278241	MK278241
*Inocybe calospora*	JFA12539WTU	Sweden	X	AY038313
*Inocybe candidipes*	BK24079907 UTC	USA: Arizona	X	AY239019
*Inocybe cerasphora*	BSI01184 WTU	Chile	X	AY380370
/Inocybe chondrospora **				
Inocybe vulpinella **	3918	China	KR082885	X
Inocybe chondrospora **	**AH34419**	**Spain**	OR088565 *	**X**
Inocybe vulpinella **	EL000610	Estonia	AM882825	X
Inocybe vulpinella **	EL18106	United Kingdom	FN550898	FN550898
Inocybe chondrospora **	**GDOR5393**	**Austria**	OR088564 *	**X**
Inocybe vulpinella **	IK-00033	Poland	KX602283	X
Inocybe vulpinella **	IK-00034	Poland	KX602282	X
Inocybe vulpinella **	IK-00061	Poland	KX602284	X
Inocybe vulpinella **	IvulpiSA01	Germany	MH389771	X
Inocybe vulpinella **	IvulpiSA02	Germany	MH389774	X
Inocybe vulpinella **	KR-M-0038284	Germany	MK929256	X
Inocybe immigrans **	**L0054131 (Isotype)**	**Canada**	MW539061 *	
Inocybe chondrospora **	**M-0151621 (Holotype)**	**Germany**	OR098441 *	OR098446 *
Inocybe vulpinella **	NI250904 (CUW)	Canada: Ontario		EU307834
Uncultured fungus **	OTU_127	Belgium	KY083605	X
Uncultured fungus **	saf_F1979	Italy: Aosta Valley	MW164608	X
Inocybe chondrospora **	**SMNS-STU-F-0901555**	**Germany**	MW539060 *	MW539060 *
Inocybe chondrospora **	**SMNS-STU-F-0901556**	**Netherlands**	MW539058 *	MW539058 *
Inocybe chondrospora **	**SMNS-STU-F-0901557**	**Germany**	MW539059 *	MW539059 *
Inocybe devoniensis **	TAA17205	Sweden	AM882826	X
Inocybe vulpinella **	UDB001758	United Kingdom	UDB001758	X
Inocybe vulpinella **	UDB017619	Norway	UDB017619	X
Inocybe vulpinella **	UDB024668	Estonia	UDB024668	X
Inocybe vulpinella **	UDB039523	Estonia	UDB039523	X
Inocybe vulpinella **	UDB0754144	United Kingdom	UDB0754144	X
*Inocybe curvipes*	PBM2401 WTU	USA: Washington	X	AY239022
*Inocybe* cf. *decemgibbosa*	G0334	Hungary	MK278224	MK278224
*Inocybe diabolica*	EL124-12	Norway	KT958913	KT958913
*Inocybe favrei*	JV30673	Norway	KY033798	KY033798
*Inocybe* aff. *fibrosoides*	TENN062452	USA: Tennessee	MT237492	MT228850
*Inocybe fibrosoides*	EL51_14	Sweden	KY033846	KY033846
*Inocybe flavobrunnescens*	EL440-13	Spain	MK153641	MK153641
*Inocybe fuligineoatra*	PBM2662 CUW	USA: Tennessee	EU523589	EU307831
*Inocybe glabrodisca*	PBM 2109 (WTU)	USA: Washington	AY239023	AY239023
*Inocybe glabrodisca*	PBM2109 WTU	USA: Washington	X	AY239023
*Inocybe hirculus*	TURA2673	Finland	MT241840	MT241840
*Inocybe inodora*	SMNS-STU-F-0901439	Austria	MT101875	MT101875
*Inocybe* aff. *insinuata*	ALW5108b (WTU)	USA: Louisiana	X	JN975030
*Inocybe intricata*	PBM2600 CUW	USA: Tennessee	EU523561	EU307835
*Inocybe krieglsteineri*	EL202-09	Sweden	KT958916	KT958916
*Inocybe kuberae*	SMNS-STU-F-0901668	Germany	ON003427	ON003427
*Inocybe lacera*	PBM1462 WTU	USA: Washington	X	AY038318
*Inocybe lanuginosa*	PBM956 WTU	USA: Washington	X	AY038319
*Inocybe lemmi*	EL106-16	Sweden	MG574395	MG574395
*Inocybe leptophylla*	BK09079719 UTC	USA: Utah	X	AY038320
*Inocybe* aff. *margaritispora*	TENN:063960/MR00198	USA: Tennessee	KP308775	JN974998
*Inocybe mixtilis ceskae*	PBM1315 WTU	USA: Washington	X	AY380387
*Inocybe napipes*	PBM2376 WTU	Norway	X	AY239024
*Inocybe nothopus*	Trappe 25060 (WTU)	Australia: New South Wales	X	AY380388
*Inocybe phaeocystidiosa*	JV29937	Sweden	MK153638	MK153638
*Inocybe phaeosticta*	PAM05072305	France	HQ586873	HQ641110
*Inocybe* cf. *praetervisa*	EL904	Sweden	AM882718	AM882718
*Inocybe* cf. *praetervisa*	SJ95027	Sweden	AM882721	AM882721
*Inocybe praetervisa*	JV23918	Italy	KY033785	KY033785
*Inocybe praetervisa*	EL8506	Sweden	FN550892	FN550892
*Inocybe praetervisa*	PBM1021 WTU	USA: Washington	X	AY038322
*Inocybe pruinosa*	SMNS-STU-F-0900987	Germany	MT101877	MT101877
*Inocybe quercicola*	HUP32966	Pakistan	MK368637	MN812170
*Inocybe rivularis*	JRJV30173	Finland	KY033813	KY033813
*Inocybe rivularis*	EL178-12	Finland	KY033823	KY033823
*Inocybe saliceticola*	BJ900827O	Sweden	AM882717	AM882717
*Inocybe salicis*	JV3319		KT958906	KT958906
*Inocybe salicis-herbaceae*	TENN:063513		X	KP170999
**/Inocybe similis**				
Inocybe similis ***	B11-9-18-1	Austria: Forchach	MT504413	X
Inocybe vulpinella ***	BR-142866-82 (Holotype)		OR098440 *	X
Uncultured Inocybe ***	MBN0213_15	Canada	KC840624	X
Inocybe similis ***	MCVE28976	Italy: Grado	KY848219	X
Inocybe similis ***	MCVE29100	Slovenia	KY848218	X
Inocybe similis ***	MCVE29287	Italy	KY848217	KY848221
Inocybe similis ***	S-F14475 (Holotype)	Italy: Trentino Alto Adige	MT704951	X
*Inocybe* sp. ***	Unknown	France	MW012258	X
Uncultured Inocybe ***	Unknown	China	OW847005	X
*Inocybe* sp.	IN36	USA: Indiana	OM473594	X
*Inocybe* sp.	JV8105		KT958910	KT958910
*Inocybe* sp.	SA100602B CUW	Slovakia	X	EU307838
*Inocybe* sp.	AJ900818		KT958923	KT958923
*Inocybe* sp.	SA100602A CUW	Slovakia	KP636858	EU307837
*Inocybe* sp.	MCA1882 WTU	Guyana	X	AY509115
*Inocybe* sp. PBM 2617	PBM 2616	USA: Tennessee	EU523567	X
*Inocybe substellata*	EL52-13	Sweden	KT958927	KT958927
*Inocybe tabacina*	PAM05071302	France	HQ586865	HQ641106
*Inocybe teraturgus*	JV4290 WTU	?	X	AY239027
Inocybe aff. *xanthomelas*	PAM07062202	France	HQ586861	HQ641104
*Inocybe* cf. *xanthomelas*	EL3505	Norway	AM882989	AM882989
*Inocybe xanthomelas*	LAS200609	Sweden	FN550895	FN550895
Uncultured fungus	clone: H226-5	China: Huayuan, Hunan	AB636455	X
Uncultured *Inocybe*	ECM_alnus_Inocvulp	France	JQ890277	X
Uncultured *Inocybe*	clone 12223_B_vivipara	Germany	KF000619	X
Uncultured *Inocybe*		China	LR598721	X
Uncultured *Inocybe*		China	LR600226	X
Uncultured *Inocybe*		China	LR818497	X
OUTGROUP				
*Inosperma* sp. NA2	PBM2871	USA: Connecticut	HQ201348	HQ201348
*Pseudosperma minutulum*	AMB 18919	Italy	ON202637	ON202637

## Data Availability

Publicly available datasets were analyzed in this study. These data can be found here: https://www.ncbi.nlm.nih.gov.

## Supplementary Materials

The following supporting information can be downloaded at: https://www.mdpi.com/article/10.3390/jof9060679/s1, Table S1: Ecological data referring to host plants of *I. chondrospora* and *I. similis*.
