# Supplementary material for: Re-Valuation of the Taxonomic Status of Species within the Inocybe similis Complex
Source: J Fungi (Basel). 2023 Jun 16;9(6):679. doi: 10.3390/jof9060679 (PMC10304480; doi:10.3390/jof9060679)
Supplement: Supplementary file 1 [file jof-09-00679-s001.zip › jof-2442257-supplementary.pdf]

Journal of Fungi

MDPI

Table S1. This is a table with ecological note about *I. similis* and *I. chondrospora.*

Species	Vouchers	Habitat/Hosts	
		Specie	plant family
Inocybe similis	B11-9-18-1	Willow tree (Salix sp.)	Salicaceae
Inocybe similis	BR-142866-82 (I. vulpinella Holotype)	Salix spp.	Salicaceae
Inocybe similis	MBN0213_15	Picea glauca	Pinaceae
Inocybe similis	MCVE28976	Populus tremula and Pinus halepensis	Salicaceae and Pinaceae
Inocybe similis	MCVE29100	Populus tremula and Salix sp.	Salicaceae
Inocybe similis	MCVE29287	Populus tremula and Pinus halepensis	Salicaceae and Pinaceae
Inocybe similis	S-F14475 (I. similis Holotype)	Populus nigra	Salicaceae
Inocybe similis	GB: MW012258	Unknown	unknown
Inocybe similis	GB: OW847005	Unknown	unknown
Inocybe chondrospora	3918	Unknown	
Inocybe chondrospora	AH34419	Salix repens cf.	Salicaceae
Inocybe chondrospora	EL000610	Unknown	unknown
Inocybe chondrospora	EL18106	Unknown	unknown
Inocybe chondrospora	GDOR5393	Salix herbacea L.	Salicaceae
Inocybe chondrospora	IK-00033	Scots pine (with alder, poplar, and willow in vicinity)	Pinaceae (Betulaceae and Salicaceae)
Inocybe chondrospora	IK-00034	Scots pine (with alder, poplar, and willow in vicinity)	Pinaceae (Betulaceae and Salicaceae)
Inocybe chondrospora	IK-00061	Scots pine (with alder, poplar, and willow in vicinity)	Pinaceae (Betulaceae and Salicaceae)
Inocybe chondrospora	IvulpiSA01	Pinus sylvestris, Salix sp.	Salicaceae and Pinaceae

Inocybe chondrospora	IvulpiSA02	Salix sp.	Salicaceae
Inocybe chondrospora	KR-M-0038284	Unknown	
Inocybe chondrospora	L0054131 (I. immigrans Isotype)	*Betula papyrifera, Populus balsamifera, Salix* sp.	Betulaceae and Salicaceae
Inocybe chondrospora	M-0151621 (I. chondrospora Holotype)	Alnus glutinosa, Salix aurita	Betulaceae and Salicaceae
Inocybe chondrospora	NI250904 (CUW)	Unknown	unknown
Inocybe chondrospora	OTU_127	*Liparis loeselii*	Orchidaceae
Inocybe chondrospora	saf_F1979	soil of alpine saffron agroecosystem (probably associated with other species not reported)	unknown
Inocybe chondrospora	SMNS-STU-F-0901555 (DB4-5-13-2)	*Salix* sp. and *Populus tremula*	Salicaceae
Inocybe chondrospora	SMNS-STU-F-0901556 (DB2-9-12-4)	*Salix repens*	Salicaceae
Inocybe chondrospora	SMNS-STU-F-0901557 (DB26-9-17-7b)	*Populus tremula*, *Salix* sp, *Pinus sylvestris*, *Betula* sp.	Salicaceae, Betulaceae and Pinaceae
Inocybe chondrospora	TAA17205	Unknown	unknown
Inocybe chondrospora	UDB001758	Salix sp.	Salicaceae
Inocybe chondrospora	UDB017619	Salix repens L.	Salicaceae
Inocybe chondrospora	UDB024668	Unknown	unknown
Inocybe chondrospora	UDB039523	Unknown	unknown
Inocybe chondrospora	UDB0754144	Unknown	unknown
